# Saw-tooth cardiomyopathy from foetal to neonatal period: a case report and literature review

**DOI:** 10.1093/ehjcr/ytaf245

**Published:** 2025-06-24

**Authors:** Zhenyu Lv, Yifei Yang, Zhiyuan Wang, Jing Yang, Yanyan Xiao

**Affiliations:** Department of Cardiology, Beijing Children’s Hospital, Capital Medical University, National Center for Children’s Health, No. 56 South Lishi Road, Beijing 100045, China; Department of Cardiology, Beijing Children’s Hospital, Capital Medical University, National Center for Children’s Health, No. 56 South Lishi Road, Beijing 100045, China; Department of Cardiology, Beijing Children’s Hospital, Capital Medical University, National Center for Children’s Health, No. 56 South Lishi Road, Beijing 100045, China; Department of Cardiology, Beijing Anzhen Hospital, Capital Medical University, No. 2 An Zhen Road, Beijing 10009, China; Department of Cardiology, Beijing Children’s Hospital, Capital Medical University, National Center for Children’s Health, No. 56 South Lishi Road, Beijing 100045, China

**Keywords:** saw-tooth cardiomyopathy, Left ventricular noncompaction, Cardiac magnetic resonance, Case report

## Abstract

**Background:**

Saw-tooth cardiomyopathy is a rare condition characterized by left ventricular (LV) dysplasia, defined by multiple myocardial crypts resembling a ‘saw-tooth’ pattern on imaging examinations.

**Case Summary:**

We present a case involving a 10-day-old neonate who was diagnosed with saw-tooth cardiomyopathy, a diagnosis substantiated by echocardiography, computed tomography (CT), and cardiac magnetic resonance imaging (CMR). The patient demonstrated arrhythmia characterized by premature atrial contractions and had a suspected cardiomyopathy identified during the foetal period.

**Discussion:**

The diagnosis of this condition is solely based on morphological features and may be misdiagnosed as LV non-compaction. Imaging modalities, including echocardiography, CT, and CMR are valuable diagnostic tools. Owing to its rarity and unclear pathogenesis, the prognosis of saw-tooth cardiomyopathy remains uncertain, necessitating long-term, potentially lifelong follow-up.

Learning points

- Saw-tooth cardiomyopathy is a rare disease characterized by left ventricular (LV) dysplasia and multiple myocardial crypts resembling a saw-tooth on imaging examinations.
- Echocardiography and cardiac magnetic resonance imaging are valuable non-invasive examinations used for the diagnosis of saw-tooth cardiomyopathy.

## Introduction

Saw-tooth cardiomyopathy is a rare condition, with only nine cases documented in the literature to date.^1^ It mainly affects the apical and middle segments of the lateral and inferior walls of the left ventricular (LV) where multiple projections of compacted myocardium resembling crypts can be observed on MRI and transthoracic echocardiography. This form of cardiomyopathy can be misdiagnosed as left ventricular non-compaction (LVNC) due to morphological similarities. We present the case of a 10-day-old neonate admitted to our hospital with arrhythmia and a suspected diagnosis of cardiomyopathy originating in the foetal period.

## Summary figure

**Figure:**
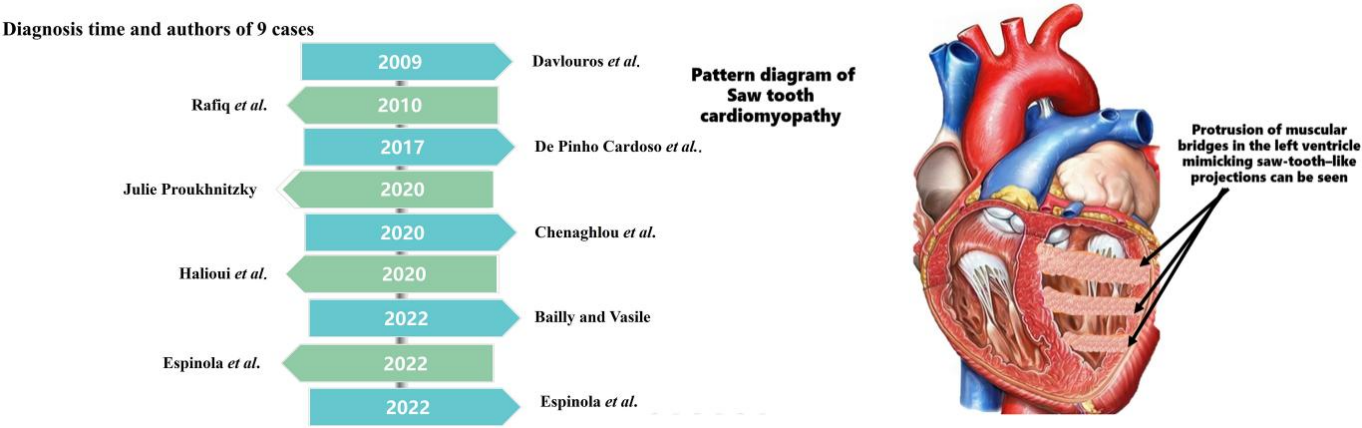


## Case presentation

**Figure 1:**
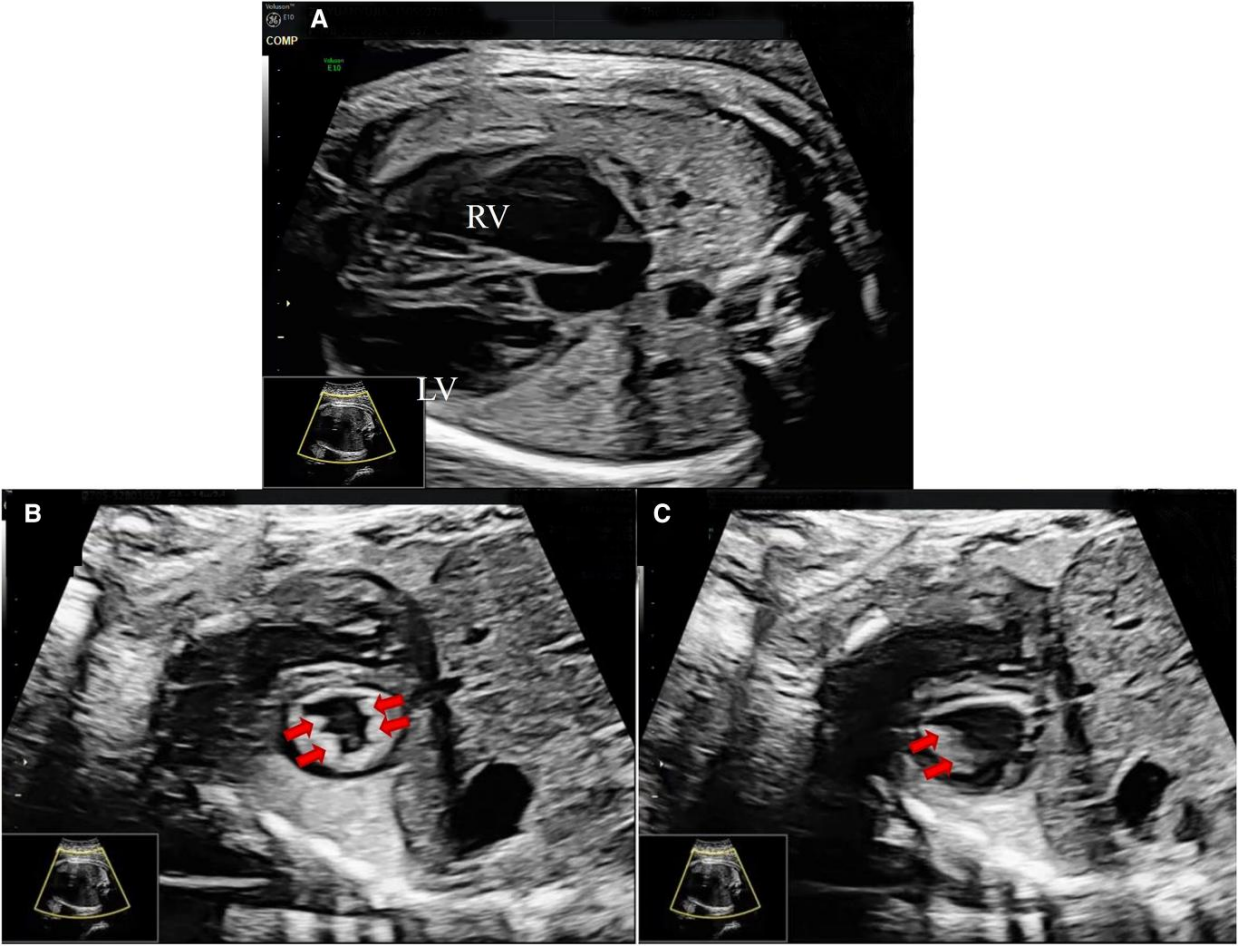
*(A)* Left ventricular enlargement. *(B)* Foetal echocardiography of the short axis in the systolic phase. Saw-tooth appearance of myocardium (arrows) in basal inferolateral and basal to mid lateral segment *(C)* Diastolic phase in a short axis section shows saw-tooth appearance of myocardium (arrows) in basal inferolateral.

**Figure 2:**
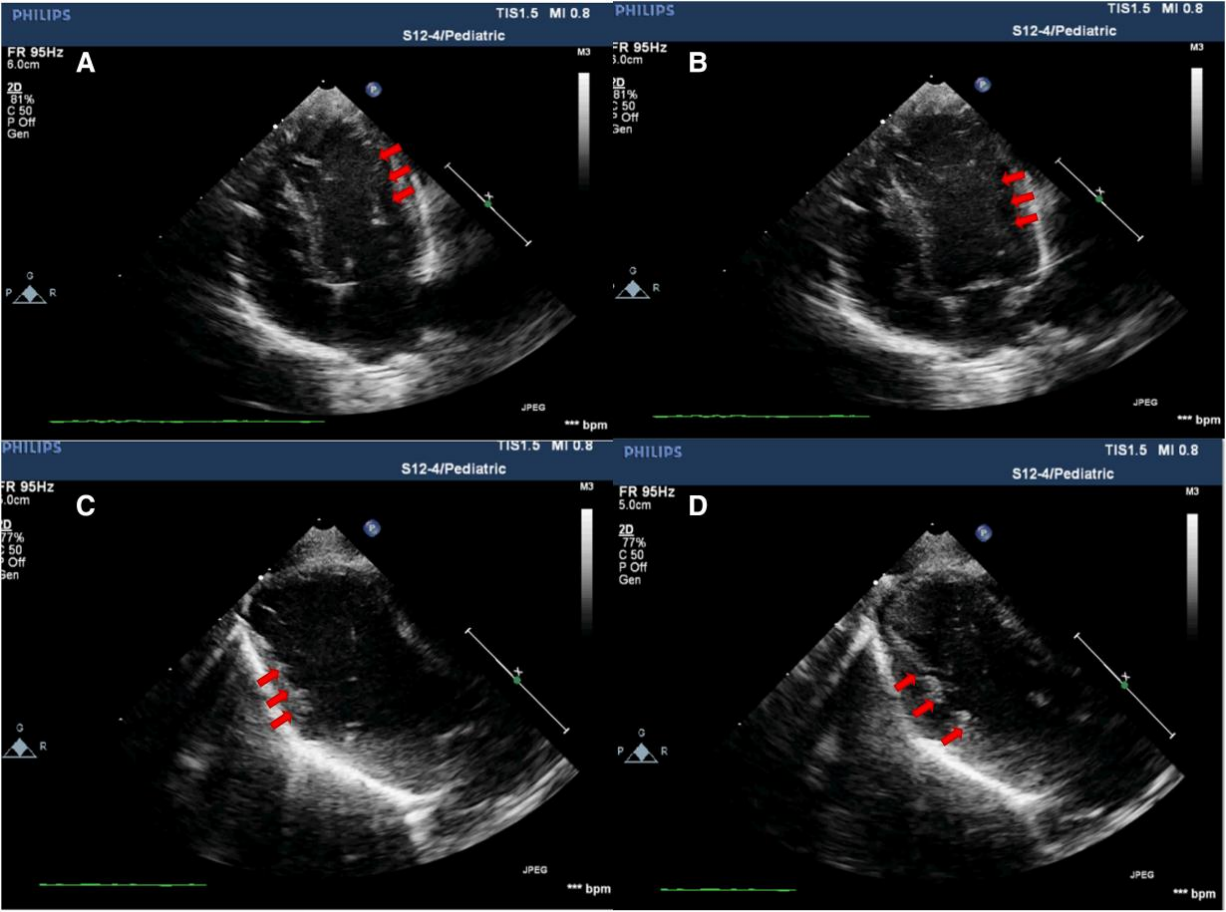
Neonatal echocardiography. (*A*) Four-chamber view of the heart in the systolic phase *(B)* Four-chamber view of the heart in the diastolic phase. *(C)* Saw-tooth cardiomyopathy is visible in the systolic period in a left ventricular long axis section (arrows). *(D)* Diastolic cross-section. There are numerous echocardiography dense saw-tooth-like inwards projections from lateral left ventricular wall and cardiac apex, clearly seen during both systole and diastole (arrows).

**Figure 3:**
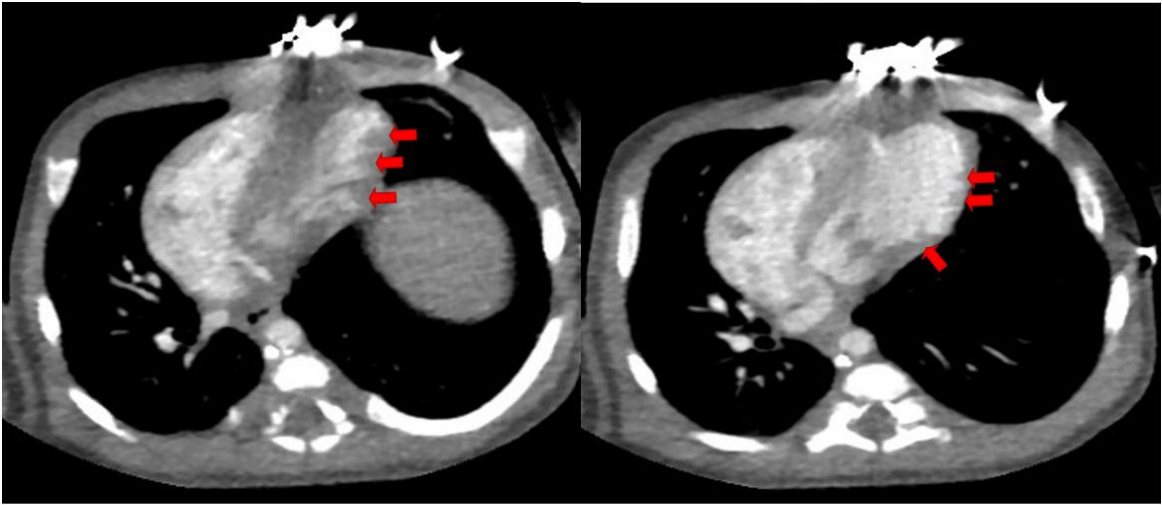
Cardiac computed tomography angiography. *(A)* A saw-tooth pattern is observed during the systolic phase (indicated by arrows). *(B)* During the diastolic phase, projections resembling a saw-tooth pattern (also indicated by arrows) can be seen.

**Figure 4:**
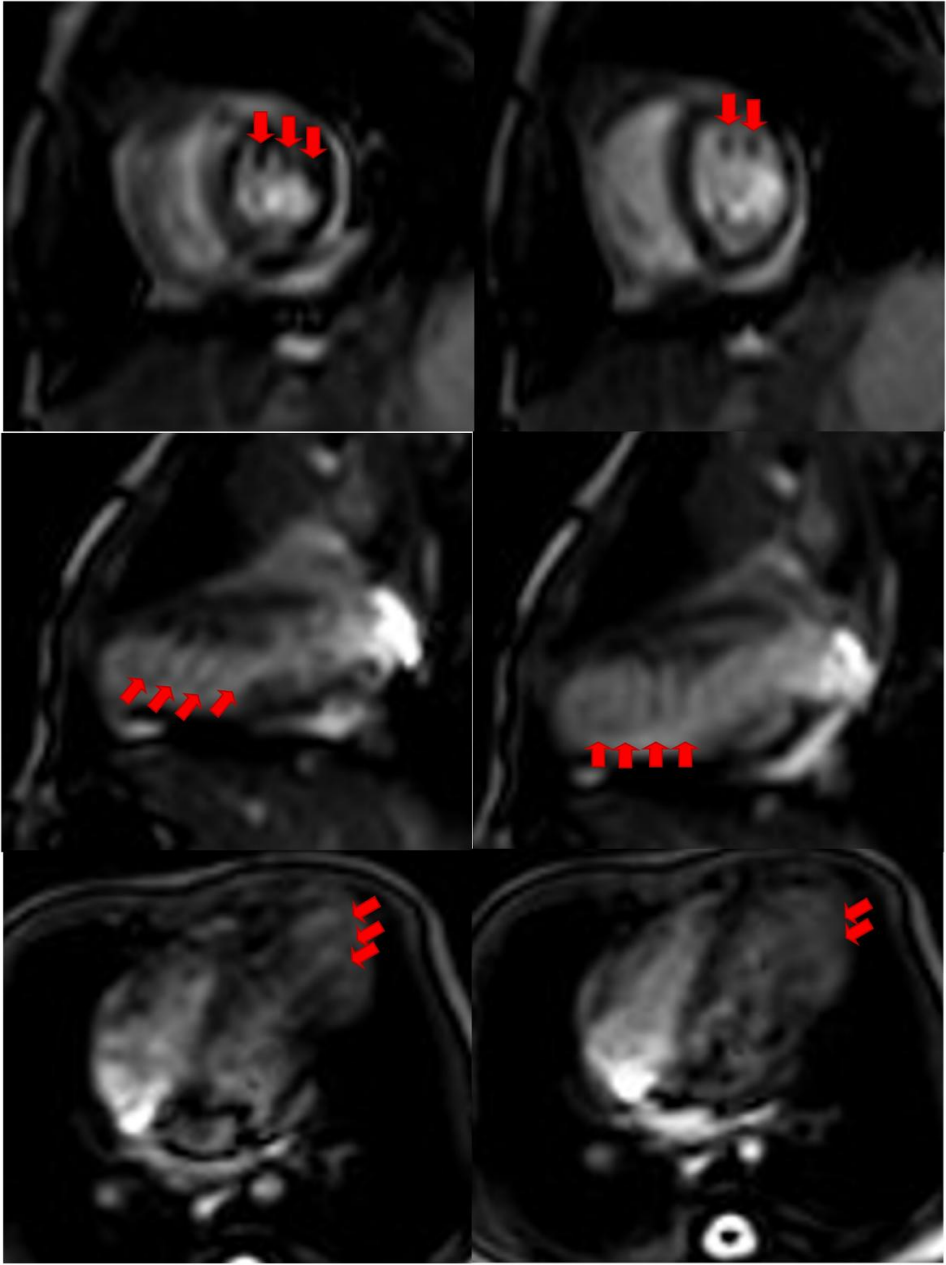
Saw-tooth cardiomyopathy is visible during the systolic phase and relaxation phase of cardiac magnetic resonance imaging. Protrusion of muscular bridges in the left ventricle mimicking saw-tooth-like projections (arrows) can be seen.

A 10-day-old female neonate, weighing 2.5 kg, was admitted to our hospital due to arrhythmia characterized by premature atrial contractions and a suspected diagnosis of cardiomyopathy identified during the foetal period. Foetal echocardiography performed at 26 weeks of gestation revealed mild LV enlargement with near-normal LV function (*Figure 1*), saw-tooth appearance of myocardium in basal inferolateral and basal to mid-lateral segment. The neonate was delivered vaginally at 36 weeks and 5 days of gestation at a local hospital, with Apgar scores of 9, 10, and 10 at 1, 5, and 10 min post-delivery, respectively. Maternal gravidity/parity history was G1P0. The mother reported no history of specific medication use during pregnancy and denied any family history of cardiomyopathy, cardiac arrest, or sudden cardiac death. At 10 days of age, the neonate was transferred to our hospital for further evaluation and management. On admission, her vital signs were stable, with 98% oxygen saturation. Respiratory auscultation revealed coarse sounds in both lungs, and the heart rate was 120 beats per min, with no detected heart murmurs. Other physical examinations were unremarkable. Laboratory tests showed normal brain natriuretic peptide levels, while Creatine Kinase MB Isoenzyme and high-sensitivity troponin I concentrations were mildly elevated. Electrocardiography revealed sinus rhythm with ST-segment elevation in leads II, III, and aVF (see Supplementary). Echocardiography (*Figure 2*) revealed mild LV apex enlargement and systolic dysfunction. An LV end-diastolic dimension (LVEDD) was within the normal range at 21 mm (*Z*-score for LV dimensions: 1.78), with an LV ejection fraction (LVEF) of 57% and fractional shortening of 30%. Additionally, hypokinesia of the ventricular septum was observed. A significant echogenic density resembling a saw-tooth pattern extended from the inferior septum and LV lateral wall into the LV lumen. The right ventricle appeared normal in size and function. Consider the LV apex dilation in the child and complete cardiac computed tomography (CT) examination. It performed with 1.0 mL/kg meglumine diatrizoate confirmed the presence of an enlarged LV apex and a saw-tooth-like protrusion, consistent with echocardiographic findings (*Figure 3*). The child’s echocardiogram suggests myocardial saw-tooth pattern; further cardiac magnetic resonance imaging (CMR) is needed for a more comprehensive evaluation. It performed with 0.1 mL/kg gadobutrol further elucidated the observed abnormalities (*Figure 4*). This imaging revealed thinning of the LV apex with outward expansion, and the local myocardium exhibited ‘serrated’ changes. The LVEF was measured at 71%, with an LVEDD of 20 mm. No surgical intervention was performed, and no medications were administered. The patient was discharged and scheduled for follow-up. At 6 months of age, the echocardiography results for the infant show findings similar to those observed previously, with normal heart function and mild LV dilation. Echocardiographic and electrocardiographic assessments of the parents were normal. A follow-up plan was established, with evaluations scheduled every 6 months.

Genetic testing revealed two heterozygous missense variants in the *MYH11* gene, *MYH11*c.4163A > Tp.E1388 V and c.487C > Tp.R163, both inherited from the mother. However, the clinical significance of these variants to cardiomyopathy remains unclear. Additionally, a heterozygous mutation of *SCN5A* c.40C > A (p.R14S) was identified, which was inherited from the father, although its association with this form of cardiomyopathy is uncertain.^2^

## Discussion

Saw-tooth cardiomyopathy is a rare condition characterized by LV dysplasia and multiple myocardial crypts, which create a saw-tooth appearance on imaging. The first case of saw-tooth cardiomyopathy was documented in 2009,^3^ and to date, only nine cases have been identified (*Table 1*). The age of onset for this condition ranges from 1 month to 75 years, with some patients exhibiting impaired ventricular function.^4^ Chest pain, frequently characterized as constrictive, represents the predominant symptom associated with saw-tooth cardiomyopathy; nonetheless, a subset of patients may present without symptoms. Diagnostic modalities such as echocardiography, CT, and CMR, have proven to be effective in the identification of this condition.^5–9^ A thorough review of the existing literature, in conjunction with our clinical observations, suggests that the LV septum is the region of the serrated myocardium most commonly affected, followed by the lateral wall.

**Table 1:** Review of cases with saw-tooth cardiomyopath

Author	Year	Country	Sex	Age	Symptoms	GA	LVEF	ECHO/CMR/Cardiac CT
Davlouros *et al*.^3^	2009	Greece	Male	2 month	No	Not done	47%	ECHO: Septal hypokinesia, LV apical aneurysm and saw-tooth morphology of the inferior, septal and lateral walls CMR: Numerous cross bridging muscular projections in the LV inferior and lateral walls and inferior inter-ven tricular septum
Rafiq *et al*.^5^	2010	UK	Male	37 years	Chestpain	Not done	Not specified	ECHO: Hypertrophic crypts at the inter-ventricular septum. CMR: Muscular bands in the inferoseptal region
De Pinho Cardoso *et al.*^6^	2017	Portugal	Male	1 month	Congestive heart failure	No pathogenic variant	Not specified	ECHO and CMR: Left ventricular dilation and apical and septal hypokinesia Numerous projections of apparently compacted myocardium originating in the LV inferior and lateral walls and on the left sur face of the inter-ventricular septum
Julie Proukhnitzky^7^	2020	France	Male	33 years	Chest pain	No pathogenic variant	55%	ECHO: showing muscular protrusions in the left cavity CMR: showing particular protrusion of muscular bridges in the left ventricle mimicking saw-tooth-like projections
Chenaghlou *et al*.^4^	2020	Iran	Male	32 years	Congestive heart failure	Not done	35%	ECHO: left ventricular enlargement CMR: Saw-tooth appearance of myocardium in basal inferolateral and basal to mid-lateral segments
Halioui *et al.*^8^	2020	Italy	Female	16 years	No	Not done	68%	CMR: Left ventricular dilation, ridges extending from the inferolateral to the inferior and inferoseptal wall
Bailly and Vasile^9^	2022	France	Male	75 years	High blood pressure	Not done	57%	CMR: Crypts at the inter-ventricular septum and left ventricular inferior wall
Espinola *et al*.^1^	2022	Mexico	Female	69 years	Chest pain, heart failure	No pathogenic variant	30%	Cardiac CT: Biventricular systolic dysfunction, no epicardial coronary lesions
Espinola *et al*.^1^	2022	Mexico	Male	49 years	No	No pathogenic variant	63%	Cardiac CT: anteroseptal middle third hypokinesia, partial calcified plaque without significant coronary obstruction

Cardiac CT, cardiac computed tomography; CMR, cardiac magnetic resonance; ECHO, echocardiography; HT, hypertension; LAD, left anterior descending; LV, left ventricle; LVEF, left ventricular ejection fraction; RVEF, right ventricular ejection fraction; GA genetic analysis.

In this case, the condition of the patient was identified prenatally, demonstrating that this condition can manifest at an early stage, even during the foetal period. Foetal echocardiography revealed LV apical enlargement and suspected cardiomyopathy; however, the serrated changes were overlooked, possibly due to the condition not being fully recognized among echocardiographers. Postnatally, the serrated changes in the LV wall were observed, distinctly differing from those in non-compaction, which are characterized by fine non-compact trabeculations and deep recesses. Given the echocardiographic findings, CMR and CT were performed to further elucidate the LV wall morphology. Compared to neonatal and foetal echocardiography, our results showed that CMR and CT provided a more detailed visualization and confirmation of the ‘saw-tooth’ morphology.

Of the nine reported cases, four underwent genetic testing. As in many other cases, no definitive pathogenic gene has been identified. Additionally, no clear genetic cause or established diagnostic criteria have been determined. The easily confused disease was non-compaction of ventricular myocardium mainly because of the confound figure. Our case demonstrates the two diseases maybe different from beginning and no evidence suggesting the same pathogenesis. The patient remains under follow-up, and no cardiac morphology or function changes have been observed. The prognosis remains uncertain owing to the rarity of the condition.

## Conclusion

We present the youngest known patient diagnosed with saw-tooth cardiomyopathy, who has been monitored from the prenatal stage to the most recent follow-up at 6 months of age. Our findings indicate that this condition may manifest as early as the foetal stage. The diagnosis relies on specific morphological features but it can be easily misdiagnosed for LVNC. Imaging modalities, including echocardiography, CT, and CMR are essential for accurate diagnosis. Owing to its rarity and unclear pathogenesis, the long-term prognosis of saw-tooth cardiomyopathy remains uncertain. Therefore, long-term, potentially lifelong follow-up is necessary.

## Supplementary Material

ytaf245_Supplementary_Data

## Data Availability

The data underlying this article will be shared upon reasonable request to the corresponding author.

## Lead author biography

Deputy Chief Physician of the Cardiology Department at Beijing Children's Hospital, Capital Medical University.

## Supplementary material

**Consent:** Informed consent for the publication of this case was obtained from the parents of the child following the Committee on Publication Ethics (COPE) guidelines.

**Funding:** None declared.
