# Supplementary figures and images for: Saw-tooth cardiomyopathy from foetal to neonatal period: a case report and literature review
Source: Eur Heart J Case Rep. 2025 Jun 24;9(7):ytaf245. doi: 10.1093/ehjcr/ytaf245 (PMC12233010; doi:10.1093/ehjcr/ytaf245)

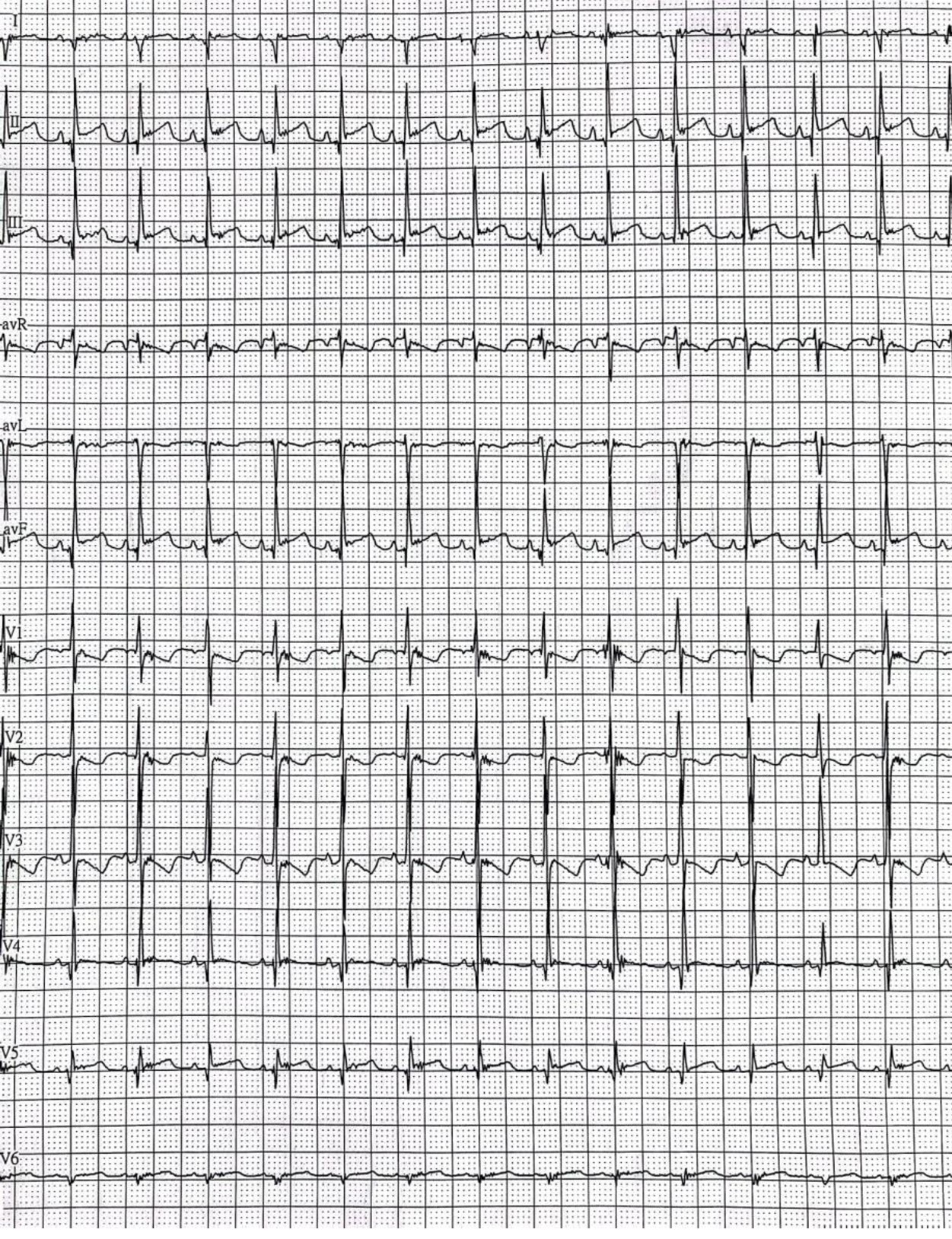

Supplement: ytaf245_Supplementary_Data [file ytaf245_supplementary_data.zip › renamed_c89c9.jpg]
